# Silver Nanoparticles Conjugated with Colistin Enhanced the Antimicrobial Activity against Gram-Negative Bacteria

**DOI:** 10.3390/molecules27185780

**Published:** 2022-09-07

**Authors:** Poowadon Muenraya, Somchai Sawatdee, Teerapol Srichana, Apichart Atipairin

**Affiliations:** 1 School of Pharmacy, Walailak University, Nakhon Si Thammarat 80161, Thailand or; 2 Drug and Cosmetics Excellence Center, Walailak University, Nakhon Si Thammarat 80161, Thailand; 3 Drug Delivery System Excellence Center and Department of Pharmaceutical Technology, Faculty of Pharmaceutical Sciences, Prince of Songkla University, Hat Yai, Songkhla 90112, Thailand

**Keywords:** silver nanoparticle, colistin, antimicrobial activity, cytotoxicity, hemolysis, characterization

## Abstract

Colistin is a potent peptide antibiotic that is effective against Gram-negative bacteria. However, nephrotoxicity limited its clinical use. Silver nanoparticles (AgNPs) have gained attention as a potential antimicrobial agent and nanodrug carrier. The conjugation of antibiotics and AgNPs has been found to increase the activity and decrease drug toxicity. In this study, colistin was conjugated with AgNPs (Col-AgNPs), which was confirmed by Fourier-transform infrared (FT-IR) and energy-dispersive X-ray (EDX) spectra. The optimized Col-AgNPs had the proper characteristics, including spherical shape, monodispersity, nanosized particle, high surface charge, and good stability. The powder X-ray diffraction (PXRD) pattern supported the crystallinity of Col-AgNPs and AgNPs. The drug loading of Col-AgNPs was 11.55 ± 0.93%. Col-AgNPs had higher activity against Gram-negative bacteria (*Escherichia coli*, *Klebsiella pneumonia*, and *Pseudomonas aeruginosa*) than AgNPs and colistin. The mechanism of actions of Col-AgNPs involved membrane disruption and genomic DNA damage. The Col-AgNPs and AgNPs were biocompatible with human red blood cells and renal cells at concentrations up to 16 µg/mL. Interestingly, Col-AgNPs exhibited higher cell survival than AgNPs and colistin at 32 µg/mL. Our results revealed that the Col-AgNPs could enhance the antimicrobial activity and cell biocompatibility more than colistin and AgNPs.

## 1. Introduction

Bacterial infection has been challenging in the healthcare system for many decades. Although researchers have discovered new antibiotics with good efficacy, bacterial diseases are still a problem worldwide because they can develop resistance to antibiotics [1]. The resistance mechanisms often involve microbial modifications to prevent drug penetration into the cells. One of the strategies to solve this problem is the combination of antibiotics with nanodrug carriers that can bring the drugs across the bacterial membrane and then facilitates the drug binding to the target sites of action. Furthermore, some drug carriers also exhibit antimicrobial activity, and they can synergize the activity when combined with antibiotics [2].

Colistin (molecular formula and mass as C_52_H_98_N_16_O_13_ and 1155.4 g/moL, respectively) is a cationic peptide antibiotic. The drug can specifically bind to lipid A of lipopolysaccharide, which is the negative charge region of the outer membrane of Gram-negative bacteria and then causes damage to the bacterial membrane, resulting in cell leakage and death [3]. However, colistin has serious adverse effects, including nephrotoxicity that depends on the dose and duration of treatment [4,5]. In addition, the drug resistance of Gram-negative pathogenic bacteria is also increasingly observed. These result in the limited use of colistin and the preservation of the drug for multidrug-resistant infections. Furthermore, the preparations of colistin in nanoformulation have been reported to enhance antimicrobial activity and reduce drug toxicity [6,7,8].

Silver nanoparticles (AgNPs) are metallic nanomaterials that show antimicrobial activity against Gram-negative and Gram-positive bacteria [9]. AgNPs are investigated in various fields for human benefits, such as antimicrobial agents, medical device coating, nanodrug carriers, cosmetic ingredients, and bioremediation [10]. The antimicrobial mechanism of AgNPs includes membrane disruption, DNA and protein destabilization, the generation of reactive oxygen species, and the inhibition of DNA replication and transcription [11]. The antimicrobial activity of AgNPs mainly employs the release of silver ions (Ag^+^) from nanoparticles. The particle size and shape are the intrinsic factors related to the surface area that determines the dissolution rate of Ag^+^ [11]. AgNPs with smaller particle sizes provide a larger surface area and exhibit more antimicrobial activity. Particle morphology also facilitates the Ag^+^ release and exerts the antimicrobial activity in which the spherical shape shows a higher killing effect than that of disk and triangle plate, respectively [12].

Many previous studies determined the activity of AgNPs when combined with antibiotic drugs, and the results showed that the activity of conjugated AgNPs was higher than that of AgNPs or free drugs alone [13]. A previous study reported the MIC value of colistin and AgNPs (5 and 215 µg/mL, respectively) against the pandrug-resistant *Acinetobacter baumannii*, and the combination between colistin and AgNPs in the ratio of 1 to 10 showed the synergistic action with an eight-time reduction in the inhibitory concentration [14]. However, that synergic study was performed by physically mixing the drug and AgNPs, and the work remained questions related to its mechanisms of action. Interestingly, the conjugation between PEGylated gold nanoparticles with colistin enhanced the target binding to the cell envelope of a Gram-negative bacterium (*Acenitobactor baumanii*) and was compatible with red blood cells [15]. In addition, AgNPs conjugated with two peptide antibiotics (colistin and bacitracin) using chemical synthesis exhibited higher antimicrobial activity against *Staphylococcus aureus* and *Escherichia coli* as the result of the increased attachment and penetration of the conjugated AgNPs into the cellular membrane. These conjugated nanoparticles also showed good biocompatibility with fibroblast cells [8].

The present study aims to develop colistin-conjugated AgNPs to improve antimicrobial activity and reduce drug toxicity. The colistin-conjugated AgNPs (Col-AgNPs) were synthesized by a chemical method in various formulations. The physicochemical properties and antimicrobial activity against Gram-negative bacteria were evaluated. The in vitro cytotoxicity of Col-AgNPs was also determined.

## 2. Results and Discussion

### 2.1. Development and Synthesis of Col-AgNPs

The synthesis of Col-AgNPs was carried out by a chemical reduction method [8,16,17]. The reducing agent was NaBH_4_, which reduced silver nitrate (AgNO_3_) to obtain AgNPs, according to this chemical reaction [18].

AgNO_3_ + NaBH_4_ → Ag + ½ H_2_ + ½ B_2_H_6_ + NaNO_3_ (1)

This current method was different from the previous studies of Mei et al. [8], Lambadi et al. [16], and Masri et al. [17], by which this method used sodium dodecyl sulfate (SDS) as a stabilizer in AgNPs [19,20,21,22]. The stabilizers have been demonstrated to play an important role in the prevention of nanoparticle agglomeration because they coat around the nanoparticle surface to protect the growth of large particles [11]. SDS functions as a stabilizer by using electrostatic repulsion, and its suitable concentration could decrease particle agglomeration [20]. According to many previous reports, the silver surface is hydrophobic and electrically negative, and the anionic SDS is adsorbed on the surface via hydrophobic bonding with a tail surface/head water configuration. The negative zeta potential of the silver nanoparticles increases as a result of SDS adsorption [20]. As a result, the silver nanoparticles are stabilized. Additionally, the presence of SDS may transform into a micellar system that inhibits AgNPs agglomeration through steric hindrance and electrical charges [22].

**Figure 1:**
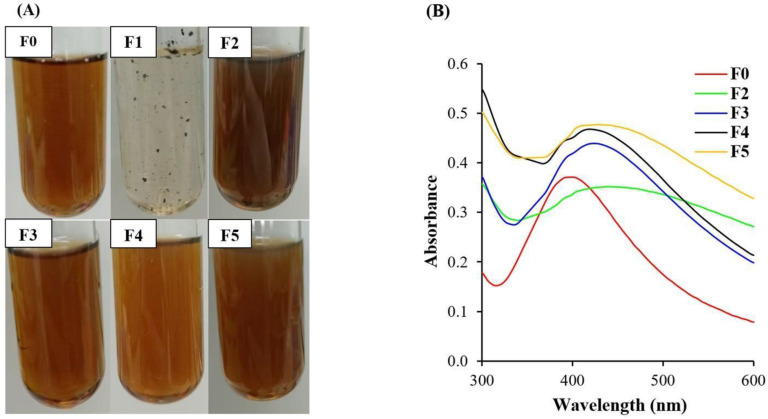
Appearance and UV-Vis spectra of nanoparticles. (**A**) The appearance of nanoparticles in the preparation of F0 (AgNPs) and F1–F5 (Col-AgNPs), and (**B**) UV-Vis spectra between 300 and 600 nm of F0 and F2–F5.

The appearances of all of the synthesized Col-AgNPs and AgNPs are shown in Figure 1A. The results of AgNP synthesis produced a brown solution (F0) that was consistent with all of the previous studies [8,16,17]. Different colors in the solutions of AgNPs may occur, such as pale yellow, golden yellow, and brownish yellow, and they depend on the concentration of the added AgNO_3_ solution. The different colors of Col-AgNPs such as pale yellow to colorless with black precipitate particles (F1), dark brown (F2, F3, F5), and dark orange to brown (F4), might be caused by the difference between SDS and colistin contents in the reactions. Col-AgNPs (F2, F3, F4, and F5) appeared as a yellow–brown liquid with transparency, so they were subjected to the next characterization. Col-AgNPs (F1) found the precipitation that might result from the lowest concentration of stabilizer (SDS) in the formulation, and it was not used for further evaluation.

### 2.2. Characteristics of Col-AgNPs

UV-Vis spectroscopy is generally used to identify the absorbance peak of silver nanoparticles as the direct consequence of the interaction of light and electrons. The light absorption and scattering properties of the nanoparticle surface affect the specific spectrum between 400 and 850 nm as the effect of particle size and shape that results in the color changes [23,24]. The UV-Vis spectra of F0 and F2–F5 showed absorbance peaks of 398 ± 0.6, 440 ± 2.0, 424 ± 1.0, 419 ± 2.5, and 430 ± 2.5 nm, and the size calculations based on the absorbance peaks according to the Mie scattering theory were 22.9 ± 0.5, 61.6 ± 1.9, 46.7 ± 0.9, 42.4 ± 2.3, and 52.6 ± 2.3 nm, respectively [25]. The increase in the particle size resulted in the shifts of the peak position to a longer wavelength that confirmed the formation of nanoparticles (Figure 1B). It was consistent with several previous studies, demonstrating that the silver nanoparticles that had a spherical shape and particle size lower than 100 nm had an absorbance peak at 400 nm, and they appeared as colloidal liquid with a yellow to brown color [8,11,16].

**Figure 2:**
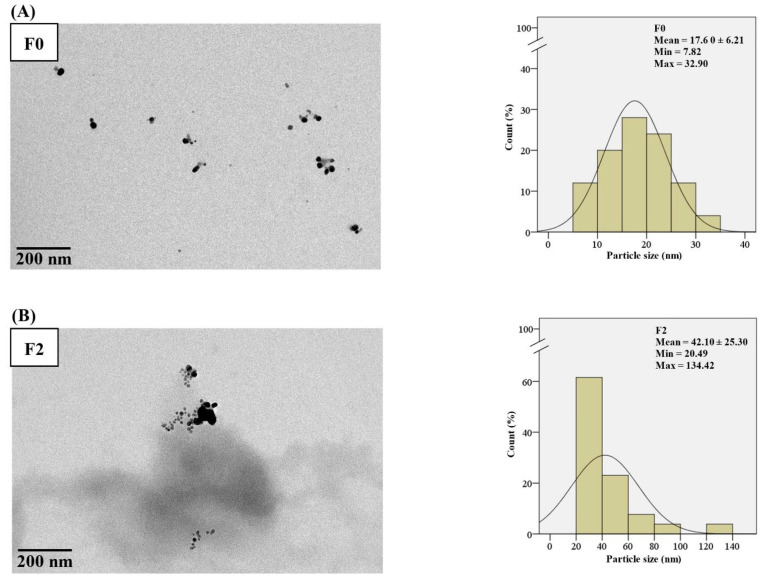

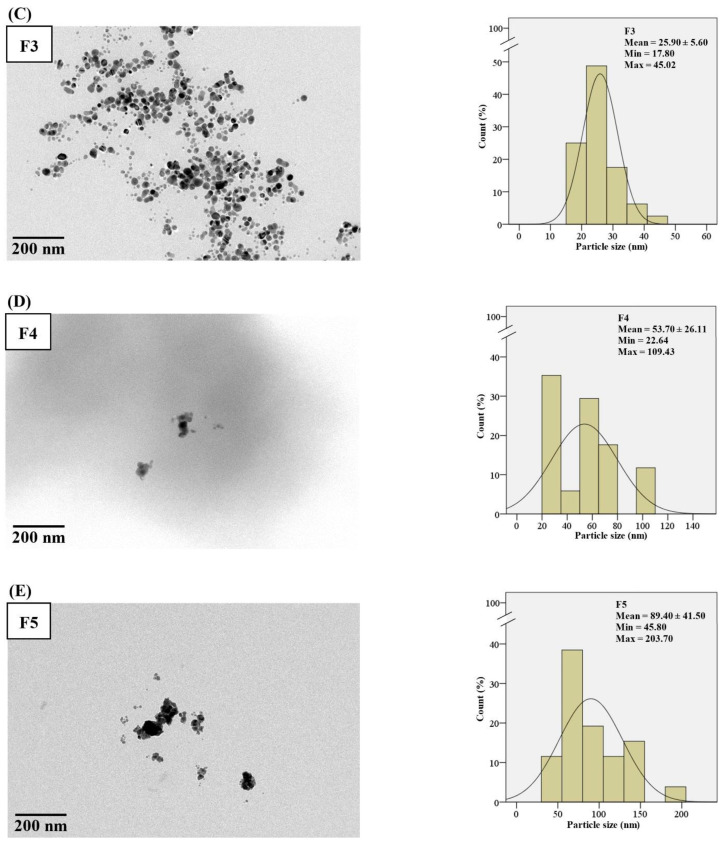
TEM micrograph and histogram of the particle size distribution of nanoparticles. (**A**) AgNPs in F0, and (**B**–**E**) Col-AgNPs in F2–F5. The electron microscope was operated at 200 kV, and the images were captured at the magnification of 40,000×. A scale bar of 200 nm was provided.

The morphology of F0, F2, F3, F4, and F5 observed by transmission electron microscope (TEM) was spherical, and their particles sizes were 17.60 ± 6.21, 42.10 ± 25.30, 25.90 ± 5.60, 53.70 ± 26.11, and 89.40 ± 41.50 nm, respectively (Figure 2). AgNPs in F0 had the smallest size and were monodispersed (Figure 2A), whereas Col-AgNPs in F2–F5 showed larger particle sizes because of the coating with colistin as the capping layer (Figure 2B–E). The particles of F2, F4, and F5 had various sizes in each formulation and formed aggregates, whereas those of F3 interestingly displayed constant size with narrow size deviation and found low aggregation. Previous studies reported a correlation between the size of the silver nanoparticles and the absorbance peak by which the increase in particle size from 10 to 90 nm resulted in the absorption change to the longer wavelength (red shift) from 400 to 420 nm [26]. It was also consistent with our study that AgNPs (F0) had a small size, showing an absorbance peak at 396 nm. Increasing the particle size of Col-AgNPs (F2–F5) caused the shift of the peak between 419 and 440 nm.

**Figure 3:**
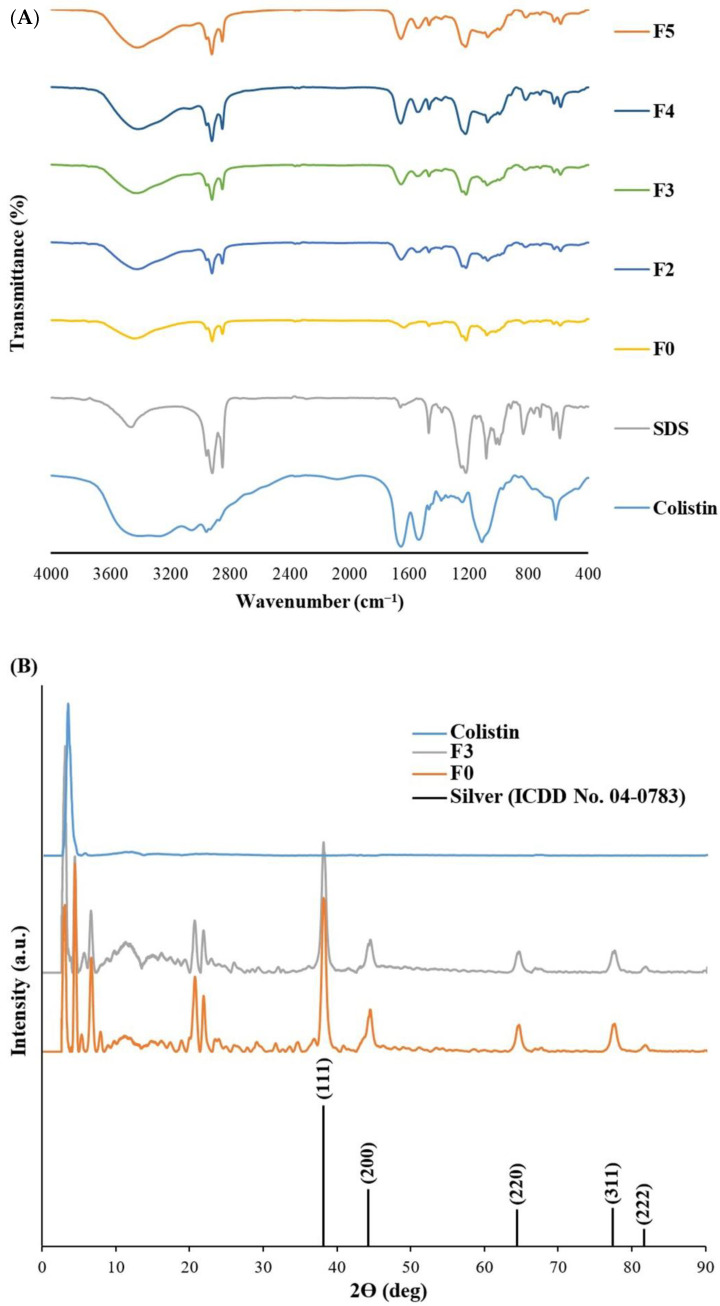
FT-IR and PXRD analysis of nanoparticles. (**A**) FT-IR spectra of colistin, SDS, AgNPs in F0, and Col-AgNPs in F2–F5, and (**B**) PXRD patterns of colistin, AgNPs in F0, and Col-AgNPs in F3.

The FT-IR spectra of F0 and F2–F5 revealed the functional groups of SDS in the nanoparticles in which the SDS signals, including S=O stretching (symmetric), S=O stretching (asymmetric), C-H stretching (symmetric), and C-H stretching (asymmetric) were found at 1219–1223, 1467–1467, 2851–2853, and 2920–2922 cm^−1^, respectively. (Figure 3A). In addition, the colistin signals in F2–F5 consisted of C-N stretching, N-H bending (amide II), C=O stretching (amide I), and C-H stretching, which occurred at 1100–1105, 1542–1548, 1654–1657, and 2957–2958 cm^−1^, respectively. These results confirmed the presence of SDS and colistin in the capping layer of nanoparticles in F2–F5.

**Figure 4:**
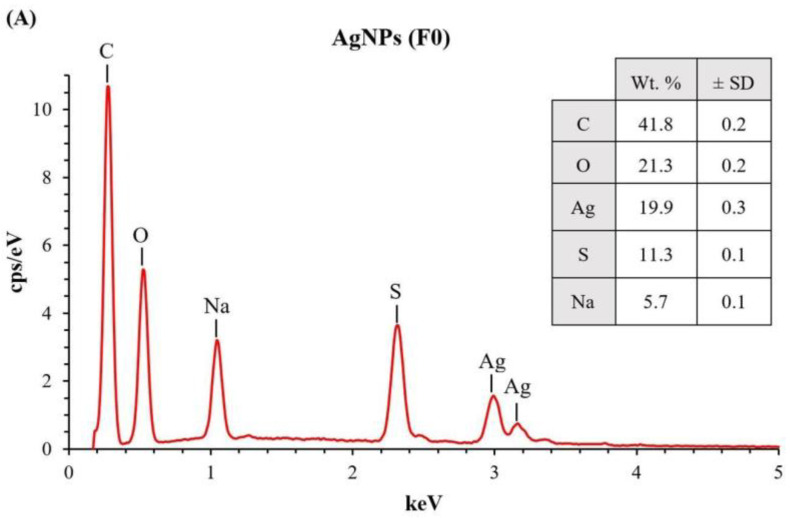

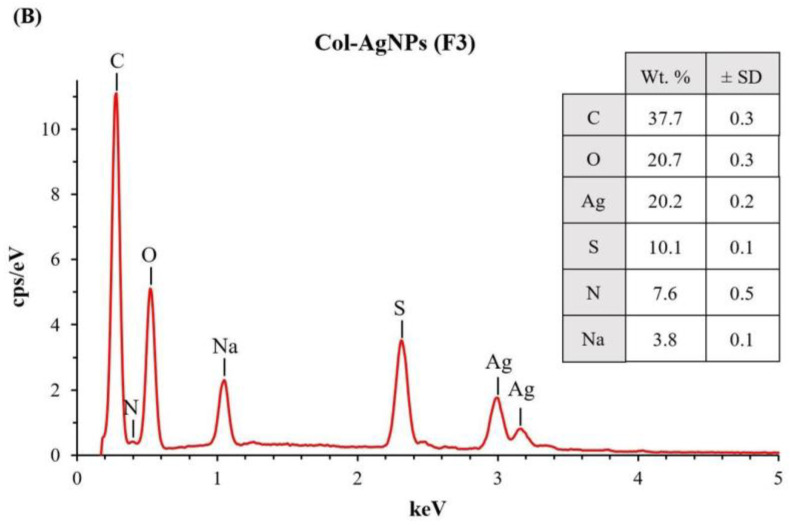
EDX spectrum of nanoparticles. (**A**) AgNPs in F0, and (**B**) Col-AgNPs in F3.

Furthermore, F3 had the smallest nanoparticles with excellent stability in size, so it was selected for powder X-ray diffraction (PXRD) and energy dispersive X-ray (EDX) spectroscopy (EDX) studies along with colistin and F0. The results revealed five distinct diffraction peaks in F0 and F3 at 2ϴ values of 38.11°, 44.42°, 64.63°, 77.58°, and 82.02° that could be indexed to the (1 1 1), (2 0 0), (2 2 0), (3 1 1), and (2 2 2) reflection planes of the face-centered cubic structure of silver (ICDD No. 04-0783) (Figure 3B) [27]. It was consistent with the PXRD pattern of AgNPs, which confirmed its crystalline form [8,28]. Colistin did not exhibit any PXRD peak because it was in an amorphous form. The peak broadening in the PXRD graphs between F0 and F3 was similar, indicating comparable crystalline domain sizes. We calculated the average crystallite size by using the Scherrer equation [29]. It was found that AgNPs (F0) and Col-AgNPs (F3) had a crystallite size of 11.47 ± 0.83 and 10.66 ± 1.33 nm, respectively. These crystalline domain sizes were smaller than the average particle size microscopically determined by TEM. It suggested that the majority of the particles in F0 and F3 were polycrystalline rather than single crystals [29]. The energy-dispersive X-ray (EDX) spectrum of Col-AgNPs and AgNPs exhibited the signal of silver element at 3 keV with a percentage of atom weight of 20.0 ± 0.2% and 19.9 ± 0.3%, respectively (Figure 4A,B). The weak nitrogen signal at 0.41 keV (7.6 ± 0.5%) was found only in the Col-AgNP sample, suggesting that it might originate from the peptide molecules of colistin that were conjugated to the surface of nanoparticles (Figure 4B). The signals of carbon, oxygen, sulfur, and sodium elements were also observed in Col-AgNPs and AgNPs, indicating the composition of coated SDS molecules.

**Figure 5:**
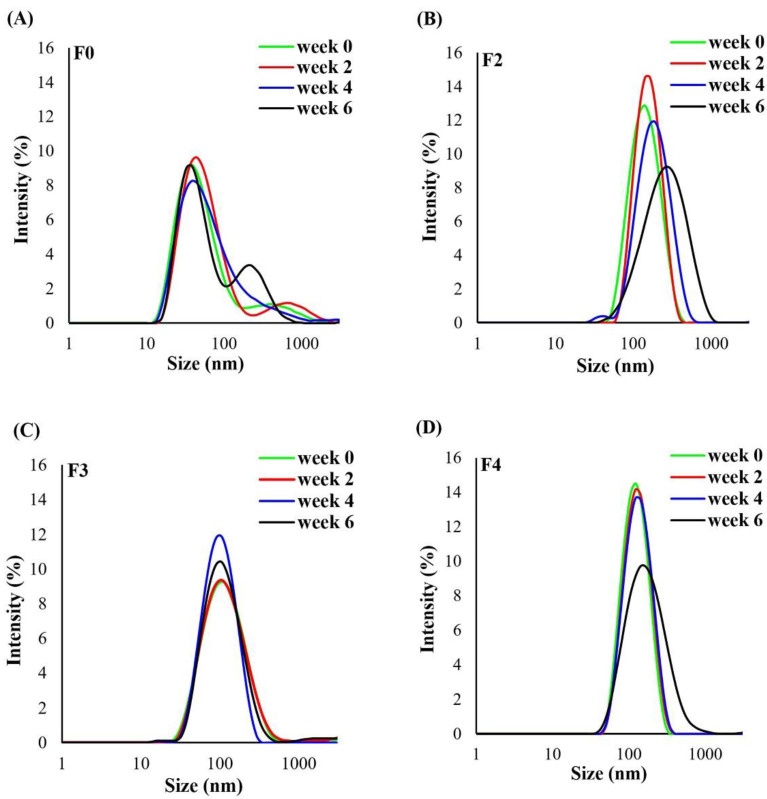

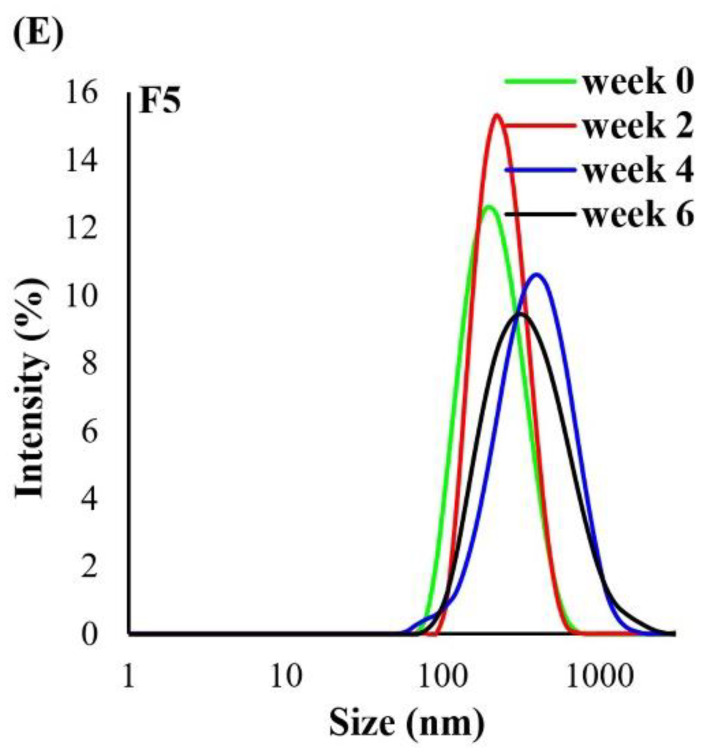
Size stability of nanoparticles. Particle size distribution of nanoparticles of (**A**) AgNPs in F0, and (**B**–**E**) Col-AgNPs in F2–F5 at week 0–6.

Table 1 shows the size of AgNPs and Col-AgNPs by dynamic light scattering (DLS) analysis. The results revealed that all of the formulations were in the nanosize ranges by which the Z-average diameters were 39.33 ± 0.01, 126.37 ± 0.42, 92.93 ± 1.48, 106.33 ± 0.21, and 198.57 ± 1.40 nm for F0, F2, F3, F4, and F5, respectively (Table 1; Figure 5). This experiment used SDS as a stabilizer that might interfere with the measurement of particle size. However, we used it at concentrations of 0.4–0.8 mM which was below the SDS critical micelle concentration (CMC) of 8 mM [30]. The SDS micelle particle has a size between 4.4 and 6.0 nm [31]. We also investigated the signal of SDS alone at the same preparation, but no observable signal was found (Figure S1). Therefore, the SDS in this study could not form a micelle and was expected to be coated with AgNP surfaces. The particle size results from DLS showed a difference from TEM analysis, which was because of the interpretation technique. The TEM analysis could directly measure the size of each particle in a small portion of the sample, while the DLS technique could do a facile analysis of the whole sample wherein the particle size was measured indirectly from light scattering. In this study, the particle size and morphology of the nanoparticles were found using TEM analysis, while the DLS analysis was used to evaluate the size stability throughout the study period.

The zeta potential of F0, F2, F3, F4, and F5 was −24.40 ± 0.26, −16.57 ± 0.06, −30.93 ± 0.85, −18.07 ± 0.31, and 16.77 ± 0.15 mV, respectively (Table 1). The high surface charge of the nanoparticles has been demonstrated to prevent the growth of larger particles because the increasing electrostatic repulsion overcomes van der Waals interaction. It also results in a decrease in the hydrodynamic size, and then the agglomeration is inhibited [32]. F0 and F3 had high surface charge based on the zeta potential, and therefore they showed no agglomeration of particles and had good size dispersibility. In contrast, F2, F4, and F5 had lower zeta potential, and then they tended to form larger flocs with large variations in particle size. These supported our result that higher size deviations based on TEM measurement and broader UV spectra were found in such formulations (F2, F4, and F5).

### 2.3. Drug Loading (DL) in Col-AgNPs

The analytical method of colistin in AgNPs was performed according to Choosakoonkriang et al., 2013 [33], the method was simple, inexpensive, and had a low detection limit for the determination of colistin. Before using this method for the assay of colistin in Col-AgNPs, we validated the method to ensure that the results were precise and accurate (Table S1). The chromatograms of colistin standard and Col-AgNPs showed the well-resolved peaks of colistin at the retention time of 9.50 min (Figure S2A,B) and the standard curve demonstrated that this analytical method had good linearity (R^2^ = 0.9997) over the concentration range of 2–150 µg/mL (Figure S2C). The accuracy (%recovery) and precision (% relative standard deviation; RSD) were 99.09 ± 0.82 and 0.50, respectively. These results showed that the analytical method was suitable for determining colistin in the silver nanoparticles system.

The %DL in the Col-AgNP formulations ranged from 9.88% to 20.08% (Table 1). F2 and F5 had high %DL when compared to other formulations, reflecting more drug coating on the Col-AgNPs. Several parameters affected the drug conjugation of the nanoparticles, including particle size, stabilizer, and drug concentration. A previous study reported that the larger nanoparticles could provide a higher capacity for drug conjugates by increasing the binding pockets for drug attachment [34]. Therefore, larger particle sizes in F2 and F5 might support higher DL of colistin in the nanoparticles. Moreover, the result also showed that an increasing amount of stabilizer (SDS) in F3 and F4 (0.8 mM), when compared with that of F2 (0.6 mM), correlated with the decrease in %DL. The unbound SDS in the formulation might interact with the positive charge of colistin, which led to the formation of strongly negative charge complexes expressed by the sulfate group of SDS (SO_4_^2^^−^) [35]. The negative charge of these complexes might prevent colistin from coating the AgNPs by electrostatic repulsion because they had the same charge as the AgNPs were capped by SDS. The highest concentration of colistin in F5 apparently resulted in the high amount of added drug to be coated on the AgNPs that caused higher %DL.

### 2.4. Stability of Col-AgNPs

The short-term stability of the nanoparticles was evaluated by storing the samples at 4 °C for the study period (6 weeks), and the nanoparticles were investigated by inspecting the appearance, measuring the particle size and zeta potential by Zetasizer, and determining the drug content using high-performance liquid chromatography (HPLC). The appearance of F0 and F2–F5 was unchanged in color, and no precipitation was observed over 6 weeks. Although their results fluctuated between the study periods, the average values of the particle size and zeta potential were gradually changed in a similar direction to the AgNPs system. The DLS analysis by Zetasizer showed that the peak positions of F0, F2, F4, and F5 shifted to a larger size, and especially there was a second peak that occurred in F0, indicating the formation of aggregates when these samples were stored for 6 weeks (Figure 5A,B,D,E). The Z-average particle size of F0, F2, F4, and F5 measured by Zetasizer at weeks 2 to 6 significantly increased when compared with that of the initial time (*p*-value < 0.05) (Table 1). Interestingly, the peaks of F3 were in the same position throughout the study period, implying this preparation had size stability (Figure 5C), and their Z-average size at weeks 2 to 6 showed no difference from that of the initial. The zeta potential value at week 6 of F0, F2, F4, and F5 slightly decreased when compared with the initial (Table 1), while the surface charge of F3 was reduced from −30.93 to −24.53 mV. These stability data, in conjunction with the zeta potential, supported the highest surface charge of F3, which could prevent particle growth (agglomeration) during 6 weeks of study. The formulation of F0 contained no colistin and could not determine the %DL. The colistin content in Col-AgNPs was investigated by a validated HPLC. The average %DL of F2–F5 at week 6 was decreased by about 4–17% of the initial content but they were not different significantly (*p*-value > 0.05). The Col-AgNPs (F5) had a high reduction in the colistin content because they had a large particle size that could accommodate a high number of peptides on their surface, giving high local peptide density [36]. The adsorbed peptides on the surface might cause structural changes, which cause them to be more susceptible to denaturation and degradation [37]. However, the long-term stability should be further investigated to obtain complete stability data. A previous study reported that colistin was stable in water for up to 60 days when stored at 4 °C [38]. In addition, the sodium deoxycholate-silver nanoparticles system was demonstrated to be stable for 7–9 months at room temperature when stored in a dark place [39].

### 2.5. Antimicrobial Activity of Col-AgNPs

The minimum inhibitory concentration (MIC) and minimum bactericidal concentration (MBC) of Col-AgNPs in F2–F5 were determined using a microdilution assay, and they were found in the range between 4.0 and 16.0 µg/mL against *E*. *coli* TISTR 887, *K*. *pneumonia* TISTR 1383, and *P*. *aeruginosa* TISTR 357. These values were lower than those of AgNPs (F0), showing bacteriostatic and bactericidal effects at concentrations of 64.0 and 128.0 µg/mL, respectively (Table 2; Figures S3–S11). These indicated that the conjugation between AgNPs and colistin exhibited higher activity than AgNPs alone as the result of the antibiotic drug that increasingly suppressed microbial growth. Col-AgNPs in F3 showed the highest inhibitory activity (4.0 µg/mL) with a broad spectrum against Gram-negative bacteria when compared with those of F2, F4, and F5 (4.0–16.0 µg/mL). Although F3 had a lesser %DL than that of F2 and F5, the strong antimicrobial activity might result from the compensation of its physical characteristics by which F3 had the smallest particle size and highest particle homogeneity. Previous studies demonstrated that the smaller particle size of AgNPs could exhibit higher antimicrobial activity [26,40]. For example, AgNPs in the size of 5 nm had higher activity than that of 15 and 55 nm against *E*. *coli*, respectively [40]. Another study presented that AgNPs (10 nm) showed the highest activity against *Vibrio natriegens* when compared with those of larger particle size (30–90 nm) [26]. In addition, our result also showed that the pure substance of colistin had the lowest MIC and MBC of 1 µg/mL, indicating that the drug was effective against these Gram-negative bacteria. The difference in the MIC and MBC values between colistin and Col-AgNPs could be explained by low drug loading in the F2–F5 formulations (9.88–20.08%).

**Figure 6:**
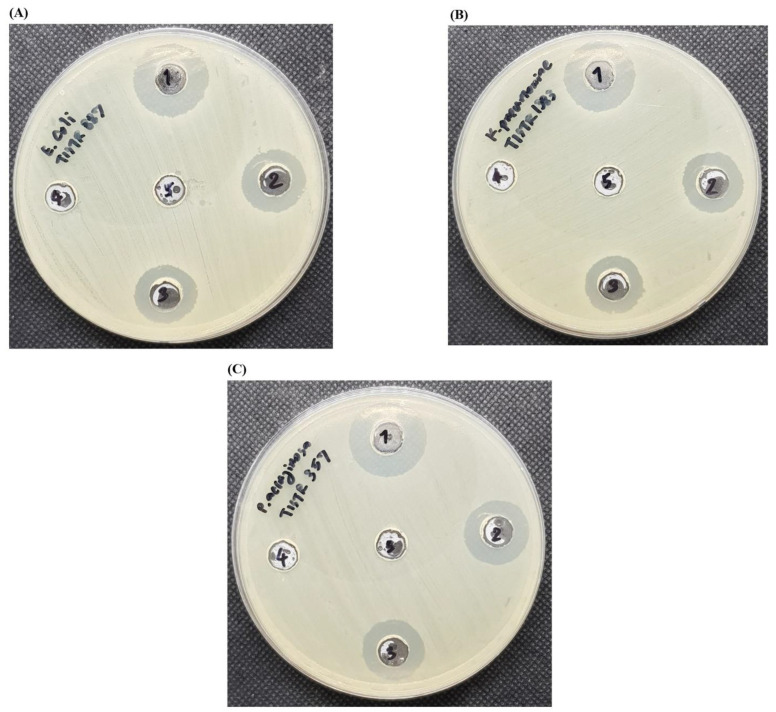
Agar well diffusion assay for nanoparticles. The indicator bacteria were (**A**) *E*. *coli* TISTR 887, (**B**) *K*. *pneumonia* TISTR 1383, and (**C**) *P*. *aeruginosa* TISTR 357. The numbers 1 to 5 represented F3, F3 physical mix, colistin, F0, and DI water, respectively.

An agar well diffusion assay was also used to determine the antimicrobial activity, and the results showed that AgNPs (64 µg/mL) in F0 provided no inhibition zone, while the preparation of F3 by physical mixing at the same concentration displayed a narrow zone toward the tested bacteria (Table 3). Col-AgNPs in F3 (64 µg/mL) had a larger inhibition zone than that of F0 and F3 physical mixing, indicating that the drug-conjugated nanoparticles exhibited more antimicrobial activity than AgNPs and non-conjugated F3 (physical mixing). The lack of active zone in AgNPs (F0) might be attributed to its concentration at MIC level, which was ineffective in killing bacterial indicators. In addition, F3 showed a greater zone of inhibition than that of colistin at the equivalent content (Figure 6A–C). It supported the enhanced antimicrobial activity of Col-AgNPs when compared with colistin and AgNPs. It could be explained that AgNPs without drug conjugation (F0) exhibited low antimicrobial activity because the negative surface charge of AgNPs expressed by SDS might interfere with the binding between the nanoparticles and the bacterial membrane, which had a negative charge. Colistin had a positive charge and interacted with high affinity to the lipopolysaccharide of the outer membrane of Gram-negative bacteria to exert its activity. When the AgNPs were conjugated with colistin (F3), colistin might predominantly mediate the nanoparticles to bind with the bacterial membrane and promote the penetration of AgNPs into the cell [41,42]. This supported the higher antimicrobial activity of the conjugated nanoparticles (F3) than that of AgNPs or colistin alone. Furthermore, the addition of AgNPs and colistin together without the conjugation (F3 physical mixing) had an activity lower than that of the conjugated AgNPs (F3) because the free colistin could not target AgNPs to bind with the bacterial membrane. It confirmed that the drug conjugation was essential for the activity. Interestingly, a recent investigation has demonstrated the synergistic activity of non-conjugated nanoparticles between green synthesized AgNPs from the leaves of *Salvia officinalis* and colistin using the disk diffusion method against multidrug-resistant bacterial pathogens [43].

### 2.6. Target Binding of Col-AgNPs

**Figure 7:**
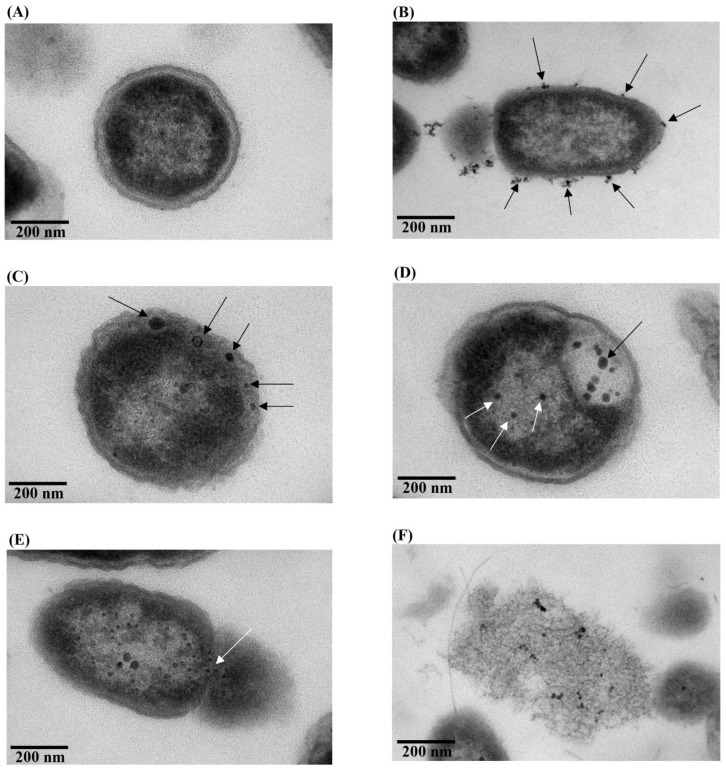
TEM micrographs of treated and untreated *P*. *aeruginosa* TISTR 357. (**A**) Untreated and (**B**–**F**) Col-AgNP-treated cells in multiple stages, covering the attachment, penetration, membrane disruption, and cell lysis.

The ultrathin section of *P. aeruginosa* TISTR 357 was observed by TEM, and it displayed an intact morphology with a thick cell wall and a smooth cell surface (Figure 7A). On the other hand, the Col-AgNPs (F3)-treated cells were monitored in many stages. Col-AgNPs could attach to the bacterial membrane in multiple sites that might be from the induction of colistin, which was coated around AgNPs because colistin was a cationic peptide molecule that specifically interacted with the negative region of the lipopolysaccharide of Gram-negative bacteria (Figure 7B) [3]. Then, Col-AgNPs could also penetrate the bacterial cell wall and destabilize the membrane (Figure 7C, black arrows), causing severe damage to the cells (Figure 7D, black arrows). Simultaneously, some particles could penetrate the cytoplasm (Figure 7D, white arrows), and the bacterial membrane had a pore that resulted in the leakage of cytoplasmic contents (Figure 7E, white arrows) and cell lysis (Figure 7F). Our result was consistent with previous studies, showing the antimicrobial mechanism of the colistin and AgNPs individually involved membrane disruption [3,11].

### 2.7. Possible Activity of Nanoparticles on the Bacterial Genomic DNA

**Figure 8:**
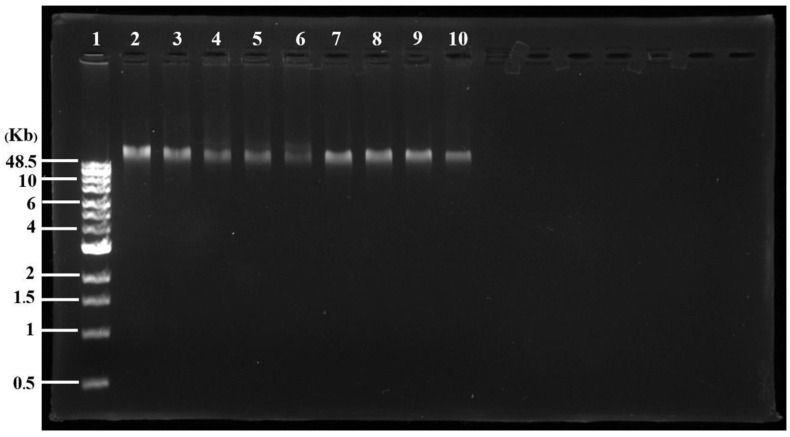
Agarose gel of treated and untreated bacterial genomic DNA. DNA marker (lane 1), untreated DNA (lane 2), and nanoparticles treated DNA with 4, 20, 40, and 80 µg/mL of Col-AgNPs (lanes 3–6) and AgNPs (lanes 7–10), respectively, were shown.

The isolated genomic DNA from *P. aeruginosa* TISTR 357 was treated with Col-AgNPs (F3) and AgNPs (F0) and analyzed on agarose gel electrophoresis. The band of the treated DNA, when increasing the concentration of the nanoparticles (Col-AgNPs and AgNPs), showed fade and smear intensity when compared to the intact band of the untreated sample (Figure 8). It confirmed the activity of the nanoparticles (Col-AgNPs and AgNPs) through the damage of the bacterial DNA. This result was supported by a previous study, demonstrating that AgNPs targeted the DNA of *P. Aeruginosa* [44]. The activity against the bacterial genome of silver nanoparticles could explain why Col-AgNPs showed better antimicrobial activity because not only did the bacterial membrane disruption cause a bactericidal effect, but they also damaged the DNA inside bacterial cells.

**Figure 9:**
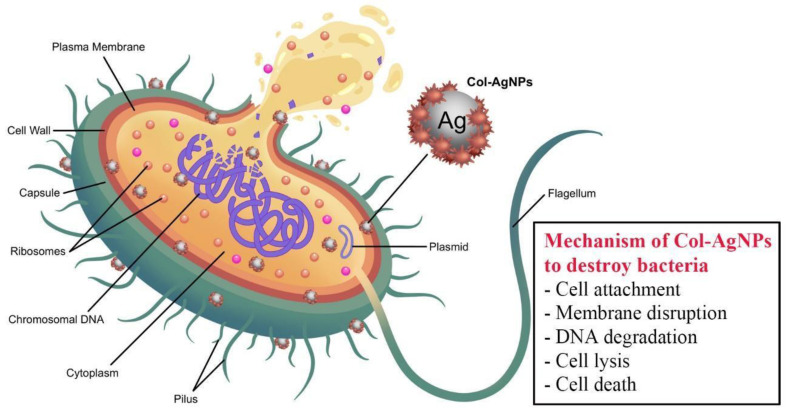
Proposed mechanism of Col-AgNPs against Gram-negative bacteria.

Taken together, the mechanism of Col-AgNPs to destroy bacteria are proposed in Figure 9. Colistin binds to lipopolysaccharides and phospholipids in the outer cell membrane of Gram-negative bacteria. It competitively replaces divalent cations (Ca^2+^ and Mg^2+^) from the phosphate groups of membrane lipids, which leads to disruption of the cell membrane, leakage of intracellular components, cell lysis, and cell death [45,46]. Silver nanoparticles could interact with the vital constituents of bacteria and can result in various damage, such as DNA degradation, destruction of cytoplasm membranes, abnormal proteins, electrolyte imbalance, and changes in gene expression, generating oxidative stress [47].

### 2.8. Hemolysis Evaluation

**Figure 10:**
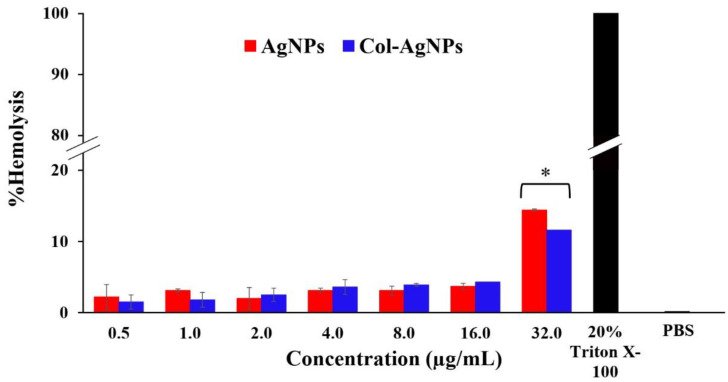
Hemolysis assay for nanoparticles. The human red blood cells were incubated with AgNPs and Col-AgNPs. 20% Triton-X 100 and phosphate-buffered saline (PBS) were used as the positive and negative controls, respectively. * indicated a significant difference at *p*-value < 0.05.

The human red blood cells were treated with Col-AgNPs (F3) and AgNPs (F0) at concentrations ranging from 0.5 to 32.0 µg/mL. The results demonstrated that Col-AgNPs and AgNPs at concentrations between 0.5 and 16.0 µg/mL were safe for human red blood cells as the %hemolysis was lower than 5.00% (1.50–4.25%) [48]. On the other hand, the %hemolysis at 32.0 µg/mL of Col-AgNPs and AgNPs increased to 11.61 ± 0.84% and 14.37 ± 1.10%, respectively, when compared to 20% Triton-X 100 as a positive control, having 100% of hemolysis. This concentration of nanoparticles (32.0 µg/mL) was considered to be toxic (Figure 10). Interestingly, there was no significant difference between Col-AgNPs and AgNPs in hemolysis at the concentrations of 0.5–16.0 µg/mL, but it was found that Col-AgNPs had significantly lower hemolysis than AgNPs at 32.0 µg/mL. A study reported that AgNPs could induce hemotoxicity in a dose-, size-, and time-dependent manner in which AgNPs at a concentration above 70 µg/mL were found to be unsafe for human red blood cells. Silver-induced hemolysis was also a function of surface area in which a large surface area provided a high dissolution rate of silver ions, and a surface area concentration of AgNPs greater than about 10 cm^2^/mL contributed to the hemolysis by the nanoparticles [49]. The possible toxicity mechanisms of AgNPs on red blood cells included the generation of silver ions, disruption of the cell membrane, releasing hemoglobin, and particle uptake [49,50].

### 2.9. Cytotoxicity of Col-AgNPs on Mammalian Cells

**Figure 11:**
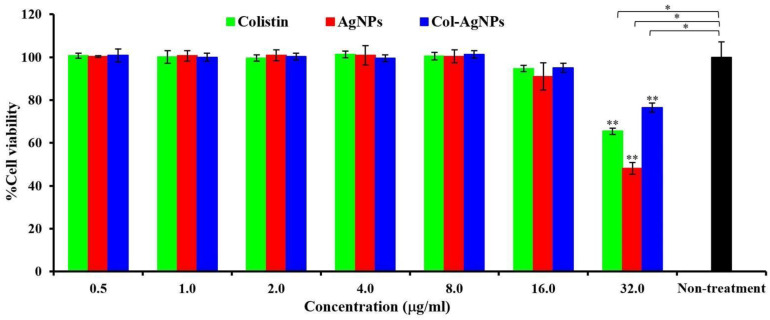
Cell viability of human primary renal proximal tubule epithelial cells. The cells were treated with colistin, AgNPs (F0), and Col-AgNPs (F3) for 24 h before determining cell viability by MTT assay. Non-treatment cells were used as the control. * and ** indicated a significant difference at *p*-value < 0.05 when compared to non-treatment and samples at the same concentration, respectively.

The cell viability of human primary renal proximal tubule epithelial cell lines in the presence of Col-AgNPs, AgNPs, and colistin was evaluated using an MTT assay (Figure 11). Colistin was biocompatible with the cell lines at 0.5–16.0 µg/mL as the cell viability was not different from the untreated cells. Col-AgNPs (F3) and AgNPs (F0) at the concentration up to 16.0 µg/mL were also biocompatible as the percentage of cell viability was insignificantly different from that of the untreated condition. The cytotoxic effect was apparently observed in silver nanoparticles and colistin at 32.0 µg/mL, showing a significant reduction in cell viability when compared to the untreated condition (*p*-value < 0.05). The result showed that the cell viability of Col-AgNPs, AgNPs, and colistin was 76.43 ± 2.18%, 48.14 ± 2.74%, and 65.44 ± 1.54%, respectively. Col-AgNPs had significantly higher cell survival, indicating that it was more compatible than AgNPs and colistin (*p*-value < 0.05). Several studies presented that the silver ions released from AgNPs could induce ROS generation in the cells, leading to lipid, protein, and DNA damage and then cell death by the apoptosis pathway [51,52]. The major factor that affects the cytotoxic degree of AgNPs is the particle size that corresponds to the surface area for ROS generation. The smaller particle size of AgNPs caused higher cytotoxicity because they could induce more ROS [53,54]. In our study, AgNPs significantly killed the cells more than Col-AgNPs at the concentration of 32.0 µg/mL. When considering the particle size of AgNPs (F0) and Col-AgNPs (F3), F0 had a smaller particle size than F3, so it had a higher surface area that might cause more ROS and cell death. The difference in capping agent or stabilizer was also reported to affect cytotoxicity, and these depended on the property and biocompatibility of the chemicals. The capped AgNPs were mostly found to have lesser cytotoxicity when compared with the uncapped AgNPs [51,53,55]. However, some capped AgNPs, such as polysaccharide-capped AgNPs, observed more cytotoxic effects on the mouse embryonic stem cells than the uncapped AgNPs [56]. The result of cell viability in this study demonstrated that colistin caused cell death at 32.0 µg/mL, and Col-AgNPs (F3) at the same concentration showed lower cytotoxicity. It was consistent with the hemolytic activity of Col-AgNPs (32.0 µg/mL), emphasizing that the silver nanoparticles were the primary factor in destroying mammalian cells. Both hemolysis and cell viability have been affected by not only the concentration of Col-AgNPs or AgNPs but also the cell concentration. Therefore, the dosing concentration that was expressed in terms of Col-AgNP or AgNP concentration per cell was determined. It was found that the increased ratio of dosing concentration correlated with cell death, and that of 3.2 × 10^4^ μg silver nanoparticles/cell significantly killed renal cells when compared to the non-treated cells. In addition, colistin was reported to cause nephrotoxicity because this drug could damage the mitochondria of kidney cells in both the dose- and time-dependent manners [3]. A previous study found that colistin (25 µg/mL) significantly induced intracellular oxidative stress at 24 h and decreased the proliferation of the immortalized proximal tubule epithelial cells (HK-2) lower than 80% [57]. Although our result found the cytotoxicity of Col-AgNPs, AgNPs, and colistin at 32 µg/mL, Col-AgNPs still provided the highest cell survival that supported the safety improvement of colistin-conjugated silver nanoparticles. The limitations of this study are the low drug loading in Col-AgNPs and the concern of nephrotoxicity in vitro at high concentrations. In addition, as the unique physicochemical characteristics of silver nanoparticles are small in size, they can pass the blood–brain barrier and cause central nervous symptoms. Recent studies demonstrated that the neurotoxicity of silver nanoparticles on a neural stem cell was not dependent on the surface charge and stabilizing agent, and its toxicity was observed at Ag concentrations of 5 μg/mL or lower [58]. However, our study did not determine the toxicity of Col-AgNPs on the neuronal cells. Therefore, it is necessary to perform further evaluations, such as the activity against drug-resistant strains, the cytotoxicity on neuronal and glial cells, and in vivo efficacy and safety of Col-AgNPs before entering the clinical study. We expect that the Col-AgNPs are the new antimicrobial compound that is beneficially used for the antibiotic-resistant crisis.

## 3. Materials and Methods

### 3.1. Materials

Colistin sulfate and sodium borohydride (NaBH_4_) were purchased from Sigma Aldrich, Inc., St. Louis, MO, USA. Sodium dodecyl sulfate (SDS), sodium sulfate, sodium phosphate dibasic, and sodium dihydrogen orthophosphate were procured from LOBA Chemie PVT. LTD., Mumbai, India. Silver nitrate (AgNO_3_), hydrochloric acid (HCl), potassium bromide (KBr), sodium chloride (NaCl), sodium hydroxide (NaOH), dimethyl sulfoxide (DMSO), and acetonitrile (ACN) were analytical grade and procured from RCI Labscan Limited, Bangkok, Thailand. Mueller Hinton (MH) and Luria Bertani (LB) agar and broth were obtained from Titan Biotech Ltd., Delhi, India. Agarose and EDTA were purchased from Bio Basic Canada Inc., Markham, Ontario, Canada. Quick-Loaded^®^ DNA ladder was from New England BioLabs Inc., Herts, England. Safe-Green dye was purchased from Applied Biological Materials Inc., Richmond, BC, Canada. E.Z.N.A.^®^ bacterial DNA kit was obtained from Omega Bio-tek Inc., Norcross, GA, USA. *E. coli* TISTR 887, *P. aeruginosa* TISTR 357, and *K. pneumonia* TISTR 1383 were from the Thailand Institute of Scientific and Technology Research, Pathum Thani, Thailand.

### 3.2. Synthesis of Colistin-Silver Nanoparticles (Col-AgNPs)

**Figure 12:**
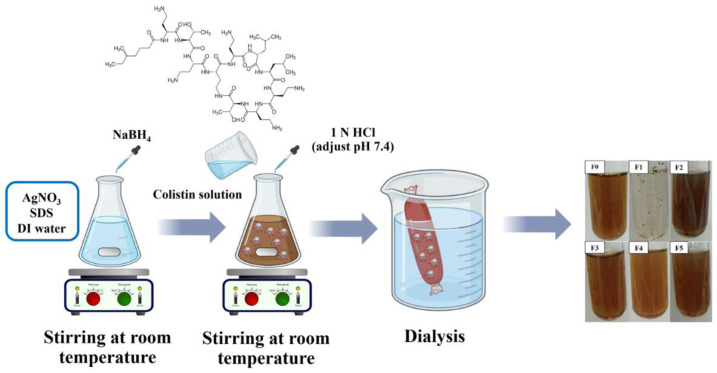
Schematic diagram of the synthesis of colistin-conjugated silver nanoparticles (Col-AgNPs).

Col-AgNPs were synthesized at room temperature by a chemical method that was slightly modified from the previous studies [8,16,17]. Our study used SDS as a stabilizer, and the reactions were performed at room temperature. The mentioned studies synthesized AgNPs coated with different antibiotics without using stabilizers and performed at room temperature or in an ice–water bath. Briefly, the concentrations of SDS as a stabilizer and colistin as an active drug were varied in the formulations (Table 4) (19). AgNO_3_ as a silver precursor and SDS were added to deionized (DI) water and mixed for 5 min. Subsequently, NaBH_4_ as a reduction agent was slowly dropped and mixed by a magnetic stirrer at 280 rpm for 10 min. The mixture was changed from transparent and colorless to brown, representing the formation of AgNPs. Colistin solution was transferred into the mixture and continuously stirred for 45 min. The pH of the mixture was adjusted to 7.4 by using 1.0 N HCl. The free ions, surfactants, and unbound drugs were removed by dialysis using a 3500 Da molecular weight cut-off membrane tubing in a large volume (1000 mL) of DI water for 24 h at room temperature under light protection. The colloidal liquid of nanoparticles was kept at 4 °C for further evaluation. In addition, the resulting colloidal Col-AgNPs after dialysis was lyophilized to remove all solvents for analysis by a freeze-drying apparatus (Christ, Martin Christ Gefriertrocknungsanlagen GmbH, Osterode am Harz, Germany). The entire synthesis of Col-AgNPs is illustrated in Figure 12.

### 3.3. Characterization of Col-AgNPs

#### 3.3.1. UV-Vis Spectroscopy

The samples (Col-AgNPs and AgNPs) were dispersed in DI water at the concentration of 50 μg/mL. The absorbance spectra were scanned in triplicate over the wavelength between 300 and 600 nm by UV-Vis spectrophotometer (Jasco Corporation, Tokyo, Japan). The scanning speed was 1000 nm/min, and the resolution was set at 1.0 nm. The 1-cm quartz cuvettes were used, and DI water was set as a blank to make the baseline correction.

#### 3.3.2. Fourier-Transform Infrared Spectroscopy (FT-IR)

The dried Col-AgNP and AgNP samples after lyophilization (1 mg) were compressed with KBr in a 1:100 ratio to obtain a 2 mm semi-transparent disk. A colistin disk was also prepared to compare the functional groups in the samples. FT-IR spectra were measured at the wavenumber of 4000–400 cm^−1^ with a resolution of 4 cm^−1^, using 16 scans/samples (Bruker Corporation, Bremen, Germany).

#### 3.3.3. Measurement of Particle Size and Zeta Potential

All of the samples (Col-AgNPs and AgNPs) were diluted with ultra-purified water at a concentration of 200 μg/mL. The particle size analysis of samples was performed by DLS using Zetasizer equipment at 25 °C (Malvern Panalytical Ltd., Malvern, UK). The particle size of all of the samples was performed at an angle of 90. The zeta potential of the samples was determined by electrophoretic mobility using Zetasizer at 25 °C, and the measurements were performed in triplicate.

#### 3.3.4. Transmission Electron Microscopy (TEM) and Energy Dispersive X-ray (EDX) Spectroscopy

Particle morphology was determined by transmission electron microscope (TEM) (JEOL Ltd., Tokyo, Japan) at 200 kV, by which the samples were dropped on the copper grid and dried under the vacuum in the desiccator. The elemental compositions of Col-AgNPs and AgNPs were investigated by an energy-dispersive X-ray spectrometer (Oxford instruments PLC, Abingdon, UK) operated at 10 kV in the scanning electron microscope (SEM). The samples of Col-AgNPs and AgNPs were fixed on the conductive carbon adhesive tape that was placed on an aluminum stub. For EDX analysis, the position of the measurement was selected in the area of the samples only without that of carbon adhesive tape.

#### 3.3.5. Powder X-ray Diffraction (PXRD)

One mg of the dried powder of Col-AgNPs (F3), AgNPs (F0), and colistin was investigated. The PXRD data were collected on a Rigaku SuperNova diffractometer with a HyPix 3000 detector using Cu Kα radiation (λ = 1.54184 Å). The diffraction patterns were collected at 25 °C and over an angular range of 0 to 90°.

### 3.4. Content of Colistin in Col-AgNPs

#### 3.4.1. Analysis of Colistin by HPLC

The colistin content in Col-AgNPs was found using HPLC (Thermo Fisher Scientific Inc., Waltham, MA, USA), according to a previous report [33]. The VertiSep^TM^ UPS C18 HPLC column (4.6 × 250 mm, 5 µm) and mobile phase (30 mM sodium sulfate pH 2.3: ACN (76: 24)) were used. The isocratic elution at a flow rate of 1.0 mL/min, a sample injection of 50 µL, and UV detector at 215 nm were applied in this chromatographic condition. The experiments were performed at room temperature. This analytical method was validated in 7 parameters, including specificity, the limit of detection (LOD), the limit of quantitation (LOQ), linearity, range, precision, and accuracy, according to The Association of Southeast Asian Nations (ASEAN) guidelines on analytical validation [59]. The standard solutions of colistin were prepared in concentrations of 2–150 µg/mL. The standard curve was obtained by plotting the graph between the concentration and area under the curve. The LOD and LOQ were calculated by multiplying a ratio of the standard deviation of the response and the slope of the standard curve by 3 and 10, respectively. Precision was measured by injecting 6 replicates of the samples and presenting the data as %RSD. Accuracy was performed by determining the % recovery of colistin at the level of 50%, 100%, and 150%.

#### 3.4.2. Drug Loading

The percentage of drug loading was evaluated by separating the free colistin and AgNPs-conjugated colistin by the dialysis method. The amount of conjugated drug was analyzed by lyophilizing 10 mL of dialyzed Col-AgNPs for 24 h and measuring the dried weight of the sample. Subsequently, the samples were dispersed with 1.3 mL of DMSO and sonicated at room temperature for 15 min. The percentage of drug loading (DL) was calculated by these equations.

(2) $$\%\mathrm{DL}\;=\frac{\mathrm{amount}\;\mathrm{of}\;\mathrm{conjugated}\;\mathrm{drug}\;\left(\mu g\right)}{\mathrm{total}\;\mathrm{amount}\;\mathrm{of}\;\mathrm{nanoparticles}\;\left(\mu g\right)}\times 100$$

### 3.5. Stability of Col-AgNPs

All of the formulations of colloidal Col-AgNPs were kept in the polyethylene tubes and stored at 4 °C for 6 weeks. The appearance, colistin content, size, and zeta potential were recorded every 2 weeks until 6 weeks. The difference in drug content, particle size, and zeta potential after storage was analyzed using the student’s t-test at the 95% confidence interval (*p*-value < 0.05). All of the experiments were performed in triplicate.

### 3.6. In Vitro Antimicrobial Activity of Col-AgNPs

The indicator bacteria, including *E. coli* TISTR 887, *P. aeruginosa* TISTR 357, and *K. pneumoniae* TISTR 1383, were cultured in MH agar at 37 °C for 24 h. The bacterial culture was adjusted to 1 × 10^8^ CFU/mL using cation-adjusted Muller–Hinton (CAMH) broth. The cell suspensions were then diluted to 5 × 10^6^ CFU/mL, and the aliquot of 10 µL was transferred into each well of a 96-well plate. One hundred µL of sample solutions containing 0.25–128 µg/mL of Col-AgNPs, AgNPs, and colistin were delivered to the wells, and the plates were incubated at 37 °C for 24 h. CAMH broth without nanoparticles and CAMH broth alone were used as the control and blank, respectively. The lowest concentration of the sample that showed no visible growth of bacteria was reported as the MIC [60]. The MBC was investigated by spreading the samples (100 µL) in each well on the MH agar. The plates were incubated at 37 °C for 24 h, and the lowest concentration of sample that showed the absence of colony was reported as the MBC. Each experiment was performed in three independent replicates.

For the agar well diffusion test, the cell suspension of the indicator bacteria (1 × 10^8^ CFU/mL) was swabbed on the MH agar, and the end of a sterile pipet tip was used to make the holes on the agar. The sample solutions (64 µg/mL) of 100 µL, including F3, F0, and a combination between AgNPs and colistin that was prepared by physical mixing equivalent to F3 (F3 mixing), were added to each well. Colistin was also used at the equivalent amount of F3, and DI water was set as a blank. The plates were incubated at 37 °C for 24 h, and each experiment was repeated in triplicate. The antimicrobial activity was evaluated by measuring the diameter of the clear zone and reported as mean ± SD.

### 3.7. Target Binding of Col-AgNPs

The cell culture (10 mL) of *P. aeruginosa* TISTR 357 (1 × 10^8^ CFU/mL) in MH broth was centrifuged at 5000× *g* for 10 min, and the pellet was washed twice with 10 mL of PBS (pH 7.4). Then, the pellet was resuspended in 10 mL of PBS, and the cells were treated with 20 µg/mL of Col-AgNPs at 37 °C for 3 h. The sample was centrifuged at 10,000× *g* for 1 min and then washed twice with PBS to remove the free nanoparticles. It was fixed with 2.5% glutaraldehyde for 2 h and washed three times with 0.1 M of phosphate buffer (pH 7.4). It was further fixed with 2% uranyl for 20 min and dehydrated by using a stepwise gradient of ethanol between 70% and 100% for 20 min. The propylene oxide and epoxy resin in ratios of 1:0, 1:1, 1:2, and 0:1 were infiltrated into samples, and they were polymerized at 70 °C for 12 h before making the cutting specimens by ultramicrotome (RMC Boeckeler Inc., Tucson, AZ, USA). The ultrathin sections were observed by TEM (JEOL Ltd., Tokyo, Japan) at 160 kV.

### 3.8. Effect of Col-AgNPs on Bacterial Genomic DNA

Several studies have demonstrated that nanoparticles could kill microorganisms by causing damage to the genomic DNA. This might be one of the modes of action of Col-AgNPs, and it was investigated by using a modified procedure [11,44]. The genomic DNA was extracted from *P. aeruginosa* TISTR 357 using an E.Z.N.A.^®^ bacterial DNA kit. Briefly, 3 mL of the bacterial suspension (1 × 10^8^ CFU/mL) was centrifuged at 10,000× *g* for 1 min and washed twice with PBS. TE buffer (100 µL) was added to resuspend the cell pellet, and the cells were lysed by lysozyme. Proteinase K and RNase were added, and the samples were centrifuged at 10,000× *g* for 2 min. The supernatant was collected and mixed with BL buffer and ethanol, respectively. The sample was transferred to a HiBind^®^ column and centrifuged at 10,000× *g* for 1 min. The column was washed with a washing buffer before eluting the DNA with sterile water (50 µL) by centrifugation at 10,000× *g* for 1 min. The bacterial genomic DNA (50 ng) was treated with Col-AgNPs and AgNPs (4, 20, 40, and 80 µg/mL) at 37 °C for 1 h. The untreated and treated DNA samples were separated on a 1% agarose gel, and then the DNA bands were visualized by Safe-Green dye. The gel was imaged by the ChemiDoc XRS+ system and analyzed by Image Lab software (Bio-Rad Ltd., Hercules, California, USA).

### 3.9. Hemolysis Evaluation

Human whole blood was collected from a healthy volunteer. The erythrocytes were obtained by centrifugation at 500× *g* for 5 min and marked the level of hematocrit (red in the lower layer) and plasma (yellowish in the upper layer) on a tube. The plasma was removed, and PBS was added to the marked line of plasma. The mixture was centrifuged at 500× *g* for 5 min and washed with PBS. The supernatant was removed and replaced with PBS. The obtained erythrocytes were dispersed in PBS at 1:50 *v*/*v*. Ten µL of stock solutions (Col-AgNPs and AgNPs) were added to the microcentrifuge tubes, and the diluted erythrocytes (190 µL) were added into each tube, giving the final concentration of nanoparticles in the range from 0.5 to 32.0 µg/mL. The tubes were incubated at 37 °C for 1 h. The negative and positive controls were PBS and 20% Triton X-100 instead of samples, respectively. The mixtures were centrifuged at 500× *g* for 5 min and 100 µL of the supernatant was transferred into a 96-well plate. The samples were measured the absorbance at 451 nm by a microplate reader. The percentage of hemolysis was calculated by this equation [61].

(3) $$\%\mathrm{Hemolysis}\;=\frac{A_{\mathrm{Sam}}-{\;A}_{\mathrm{Neg}}}{A_{\mathrm{Pos}}-{\;A}_{\mathrm{Neg}}}\times 100$$

where A_Sam_, A_Neg_, and A_Pos_ were the absorbance of sample, negative control, and positive control, respectively.

### 3.10. Cell Viability Test by MTT Assay

Human primary renal proximal tubule epithelial cell lines (PCS-400-010, ATCC, Manassas, VA, USA) were cultured in the renal epithelial cell basal medium supplemented with a renal epithelial cell growth kit. The cells were incubated at 37 °C in a 5% CO_2_ incubator, and the media was changed every 2 days. When the cells reached 80% confluence, they were harvested using 0.25% trypsin-EDTA (Gibco^®^, Grand Island, NY, USA), followed by the addition of a fresh culture medium. The cells at a concentration of 1 × 10^5^ cells/mL were seeded in a 96-well plate and incubated at 37 °C under 5% CO_2_ for 24 h. The samples of various concentrations of Col-AgNPs and AgNPs (0.5, 1, 2, 4, 8, 16, 32, 64, and 128 µg/mL) in a fresh medium were added into the culture plates. The cells without samples served as a negative control. After incubation for 24 h, a methylthiazol tetrazolium (MTT) assay was performed to evaluate cell viability. Briefly, the cells were treated with 80 µL of fresh media along with 20 µL of MTT solution and incubated at 37 °C under 5% CO_2_ for 4 h. Thereafter, media containing MTT were removed, and 100 µL of DMSO was added. The absorbance at 570 nm was determined by a microplate reader (Biohit Oyj, Helsinki, Finland). The percentage of cell viability was calculated by comparing the absorbance between the treated and untreated samples.

## 4. Conclusions

Col-AgNPs were synthesized by a chemical method, and it was found that the concentrations of colistin and SDS affected the physicochemical properties, including surface plasmon resonance, particle size, surface charge, and drug content. Col-AgNPs in F3, which had a small particle size and a high surface charge, showed great stability in the study period, and the diffraction pattern supported its formation of a crystalline structure. The Col-AgNPs exhibited higher antimicrobial activity against Gram-negative bacteria than AgNPs and colistin, by which they attached and destroyed the bacterial membrane before penetration inside the cells, leading to the leakage of cytoplasmic content. Both Col-AgNPs and AgNPs also damaged the bacterial genomic DNA. The safety profile of Col-AgNPs revealed that it was biocompatible with human red blood cells and human primary renal proximal tubule epithelial cells at concentrations up to 16.0 µg/mL. At higher concentrations (32.0 µg/mL), Col-AgNPs significantly caused lower mammalian cell death when compared to AgNPs and colistin. It would provide an opportunity for the development of a potent antimicrobial agent with reduced cytotoxicity to be safe for human therapy.

## Figures and Tables

**Table 1:** Particle size, zeta potential, and colistin content of AgNPs (F0) and Col-AgNPs (F2–F5). The results after storage at 4 °C for 6 weeks were determined, and they were expressed in mean ± SD; *n* = 3.

Formulation Code	Initial	Week 2	Week 4	Week 6
**Size (Z–average; d.nm)**				
F0	39.33 ± 0.01	43.10 ± 0.28 *	44.91 ± 0.07 *	51.01 ± 0.20 *
F2	126.37 ± 0.42	142.53 ± 2.14 *	162.50 ± 1.22 *	217.00 ± 0.85 *
F3	92.93 ± 1.48	91.23 ± 1.23	93.59 ± 1.87	94.95 ± 4.65
F4	106.33 ± 0.21	114.43 ± 0.51 *	118.57 ± 0.12 *	145.03 ± 0.47 *
F5	198.57 ± 1.40	222.63 ± 6.21 *	331.57 ± 0.67 *	300.83 ± 1.45 *
**Zeta potential (mV)**				
F0	−24.40 ± 0.26	−23.20 ± 0.45	−23.77 ± 0.38 *	−22.20 ± 0.46 *
F2	−16.57 ± 0.06	−16.47 ± 0.40	−15.94 ± 0.27 *	−15.77 ± 0.06 *
F3	−30.93 ± 0.85	−22.90 ± 1.18 *	−22.10 ± 0.36 *	−24.53 ± 0.90 *
F4	−18.07 ± 0.31	−19.67 ± 0.32 *	−19.17 ± 0.80	−17.37 ± 0.40
F5	−16.77 ± 0.15	−17.23 ± 0.38	−16.73 ± 0.55	−15.03 ± 0.15 *
**Colistin content %**				
F0	ND	ND	ND	ND
F2	19.85 ± 1.24	20.05 ± 1.36	19.22 ± 1.22	18.44 ± 1.62
F3	11.55 ± 0.93	10.54 ± 0.41	10.52 ± 0.61	10.36 ± 0.22
F4	9.88 ± 0.65	10.12 ± 0.59	9.81 ± 0.38	9.52 ± 0.22
F5	20.08 ± 1.79	19.64 ± 1.86	18.04 ± 2.13	16.72 ± 2.39

ND = not determined; * indicated a significant difference at *p*-value < 0.05.

**Table 2:** Determination of MIC and MBC value. MIC and MBC of colistin, AgNPs in F0, and Col-AgNPs in F2–F5 against *E. coli* TISTR 887, *K. pneumonia* TISTR 1383, and *P. aeruginosa* TISTR 357.

Bacterial Indicators	MIC/MBC (µg/mL)					
	Colistin	F0	F2	F3	F4	F5
*E. coli* TISTR 887	1.0/1.0	64.0/128.0	16.0/16.0	4.0/4.0	8.0/16.0	8.0/8.0
*K. pneumonia* TISTR 1383	1.0/1.0	64.0/128.0	8.0/8.0	4.0/4.0	8.0/8.0	4.0/4.0
*P. aeruginosa* TISTR 357	1.0/1.0	64.0/128.0	4.0/4.0	4.0/4.0	4.0/4.0	4.0/4.0

**Table 3:** Agar well diffusion assay for nanoparticles. Inhibition zone of colistin, AgNPs in F0, and Col-AgNPs in F3 and F3 physical mixing against *E. coli* TISTR 887, *K. pneumonia* TISTR 1383, and *P. aeruginosa* TISTR 357. The result was presented as mean ± SD; *n* = 3.

Bacterial Indicators	Diameter of Inhibition Zone			
	Colistin (7.4 μg/mL)	F0 (64 μg/mL)	F3 (64 μg/mL)	F3 Physical Mixing (64 μg/mL)
*E. coli* TISTR 887	15.23 ± 0.25	0.00 ± 0.00	18.40 ± 0.10	16.23 ± 0.06
*K. pneumonia* TISTR 1383	15.27 ± 0.25	0.00 ± 0.00	18.40 ± 0.10	16.20 ± 0.30
*P. aeruginosa* TISTR 357	15.70 ± 0.10	0.00 ± 0.00	19.33 ± 0.12	16.37 ± 0.32

**Table 4:** Formulations of Col-AgNPs and AgNPs.

Formulation Code	AgNO_3_ (mM)	NaBH_4_ (mM)	SDS (mM)	Colistin (µg/mL)	DI Water (mL)
F0	0.33	2.0	0.8	0	50
F1	0.33	2.0	0.4	50	50
F2	0.33	2.0	0.6	50	50
F3	0.33	2.0	0.8	50	50
F4	0.33	2.0	0.8	75	50
F5	0.33	2.0	0.8	100	50

## Data Availability

Not applicable.

## Supplementary Materials

The following supporting information can be downloaded at: https://www.mdpi.com/article/10.3390/molecules27185780/s1, Figure S1: Particle size distribution of SDS alone (0.8 mM) at the same preparation; Figure S2: HPLC chromatograms and standard curve of colistin; Figure S3: MIC of colistin and F0 against *E*. *coli* TISTR 887; Figure S4: MIC of F2 and F3 against *E*. *coli* TISTR 887; Figure S5: MIC of F4 and F5 against *E*. *coli* TISTR 887; Figure S6: MIC of colistin and F0 against *K*. *pneumonia* TISTR 1383; Figure S7: MIC of F2 and F3 against *K*. *pneumonia* TISTR 1383; Figure S8: MIC of F4 and F5 against *K*. *pneumonia* TISTR 1383; Figure S9: MIC of colistin and F0 against *P*. *aeruginosa* TISTR 357; Figure S10: MIC of F2 and F3 against *P*. *aeruginosa* TISTR 357; Figure S11: MIC of F4 and F5 against *P*. *aeruginosa* TISTR 357; Table S1: Summary of the parameters from the validated HPLC method for determining colistin in Col-AgNPs.
