# Supplementary material for: Silver Nanoparticles Conjugated with Colistin Enhanced the Antimicrobial Activity against Gram-Negative Bacteria
Source: Molecules. 2022 Sep 7;27(18):5780. doi: 10.3390/molecules27185780 (PMC9505607; doi:10.3390/molecules27185780)
Supplement: Supplementary file 1 [file molecules-27-05780-s001.zip › molecules-1898381-supplementary.pdf]

Supplementary Materials: Silver Nanoparticles Conjugated with Colistin Enhanced the Antimicrobial Activity against Gram-Negative Bacteria

Poowadon Muenraya [1,2], Somchai Sawatdee [1,2], Teerapol Srichana [3] and Apichart Atipairin [1,2,*]

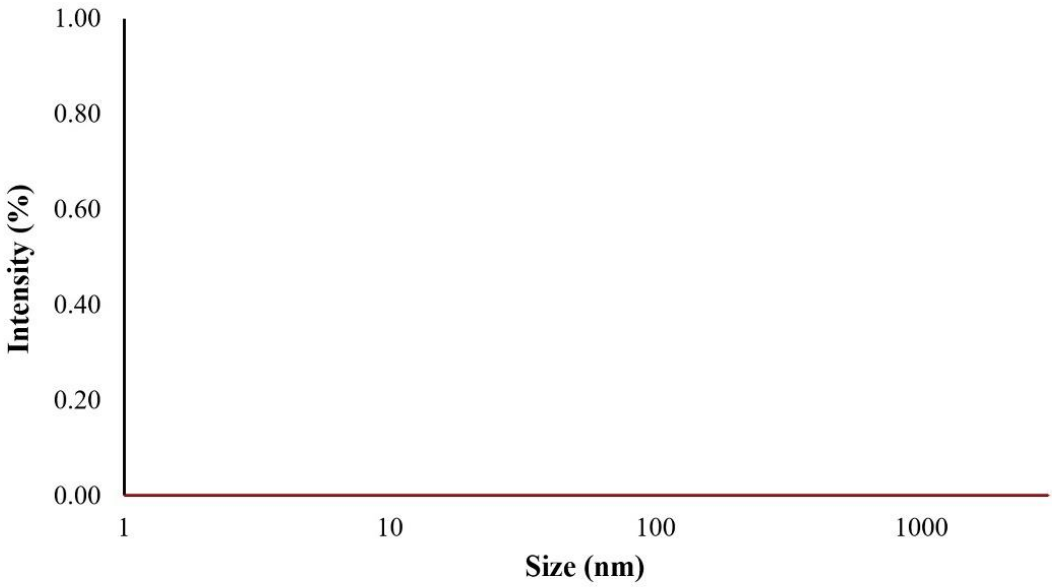

Figure S1. Particle size distribution of SDS alone (0.8 mM) at the same preparation. SDS signal was in the red line.

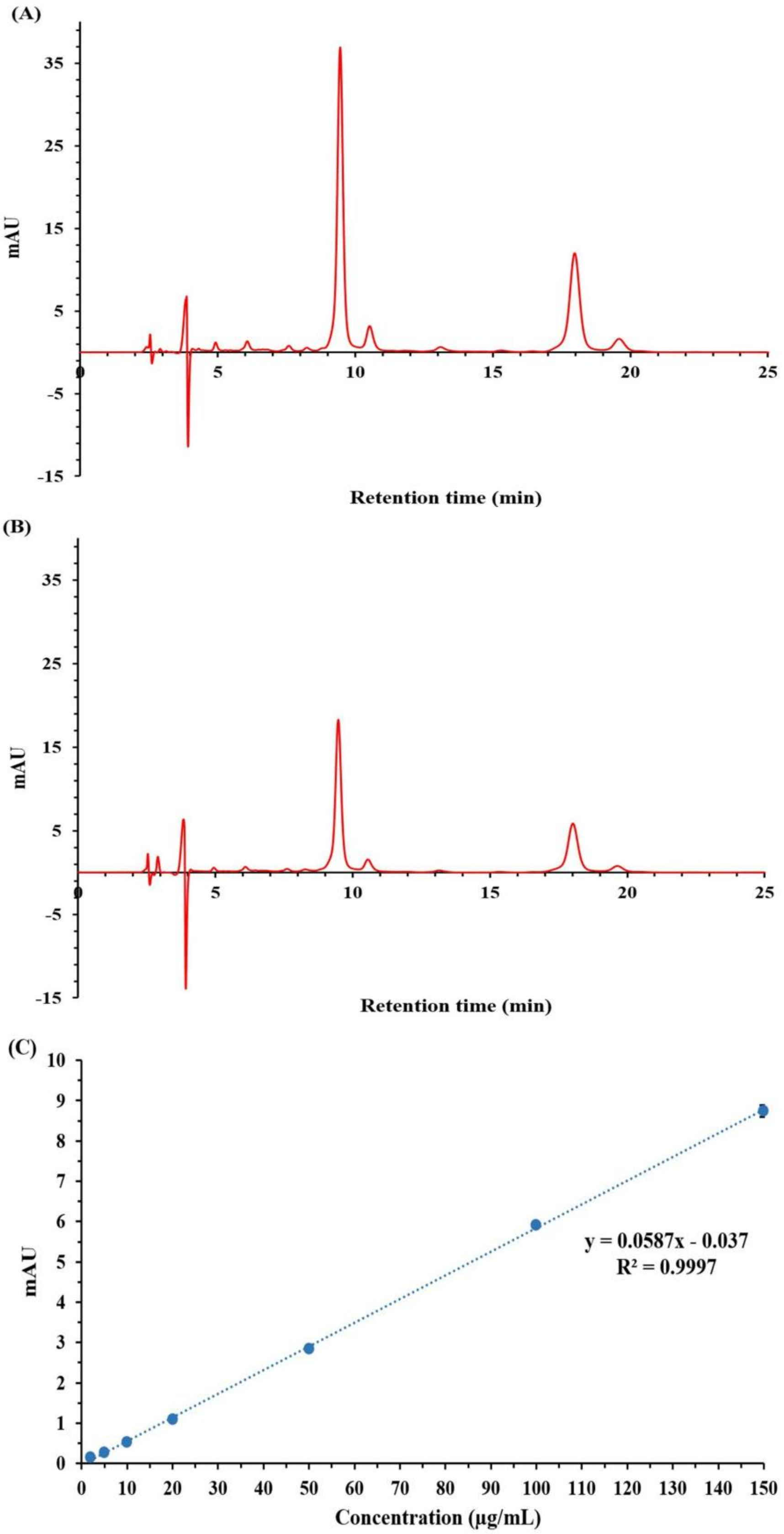

Figure S2. HPLC chromatograms of (A) colistin standard and (B) colistin in Col-AgNPs. (C) The standard curve of colistin standards at 2, 5, 10, 20, 50, 100, and 150 μg/mL.

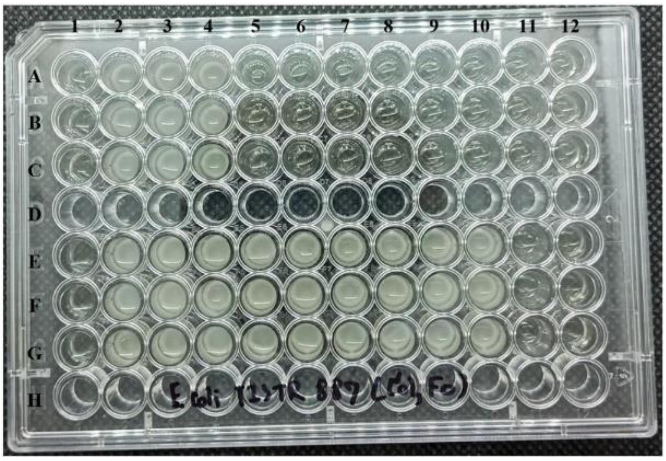

Figure S3. MIC of colistin and AgNPs in F0 against *E. coli* TISTR 887. Blank (wells 1A-1C and 1E-1G), untreated (wells 2A-2C and 2E-2G), colistin at 0.25, 0.5, 1, 2, 4, 8, 16, 32, 64, and 128 μg/mL (wells 3A-3C – 12A-12C, respectively), and AgNPs in F0 at 0.25, 0.5, 1, 2, 4, 8, 16, 32, 64, and 128 μg/mL (wells 3E-3G – 12E-12G, respectively).

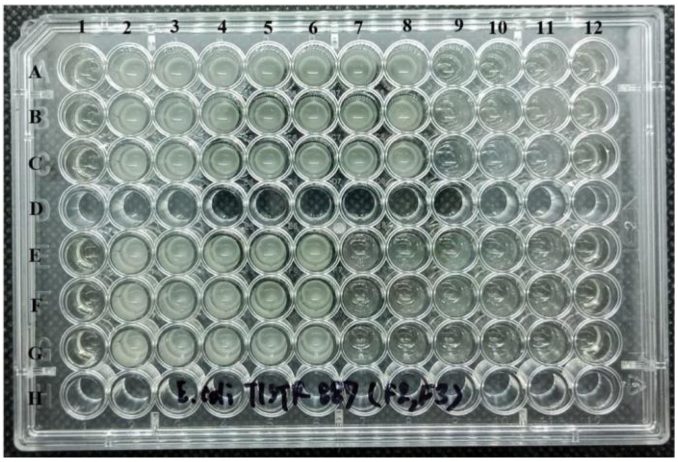

Figure S4. MIC of Col-AgNPs in F2 and F3 against *E. coli* TISTR 887. Blank (wells 1A-1C and 1E-1G), untreated (wells 2A-2C and 2E-2G), Col-AgNPs in F2 at 0.25, 0.5, 1, 2, 4, 8, 16, 32, 64, and 128 μg/mL (wells 3A-3C – 12A-12C, respectively), and Col-AgNPs in F3 at 0.25, 0.5, 1, 2, 4, 8, 16, 32, 64, and 128 μg/mL (wells 3E-3G – 12E-12G, respectively).

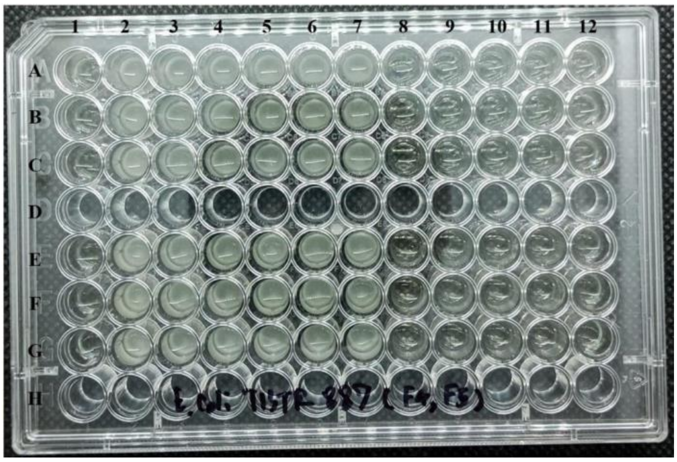

Figure S5. MIC of Col-AgNPs in F4 and F5 against *E. coli* TISTR 887. Blank (wells 1A-1C and 1E-1G), untreated (wells 2A-2C and 2E-2G), Col-AgNPs in F4 at 0.25, 0.5, 1, 2, 4, 8, 16, 32, 64, and 128 μg/mL (wells 3A-3C – 12A-12C, respectively), and Col-AgNPs in F5 at 0.25, 0.5, 1, 2, 4, 8, 16, 32, 64, and 128 μg/mL (wells 3E-3G – 12E-12G, respectively).

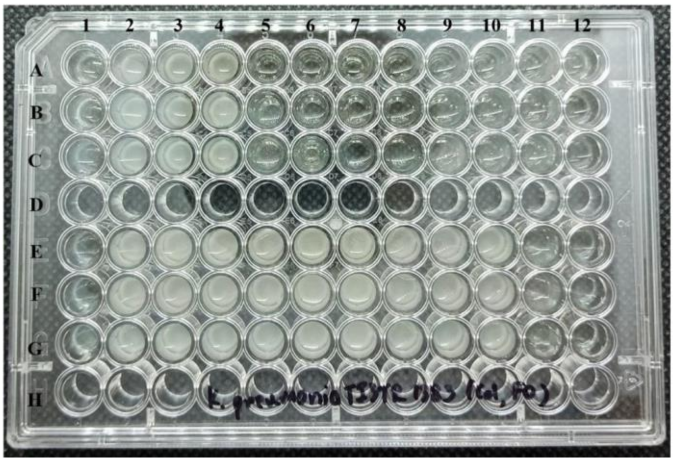

Figure S6. MIC of colistin and AgNPs in F0 against *K. pneumonia* TISTR 1383. Blank (wells 1A-1C and 1E-1G), untreated (wells 2A-2C and 2E-2G), colistin at 0.25, 0.5, 1, 2, 4, 8, 16, 32, 64, and 128 μg/mL (wells 3A-3C – 12A-12C, respectively), and AgNPs in F0 at 0.25, 0.5, 1, 2, 4, 8, 16, 32, 64, and 128 μg/mL (wells 3E-3G – 12E-12G, respectively).

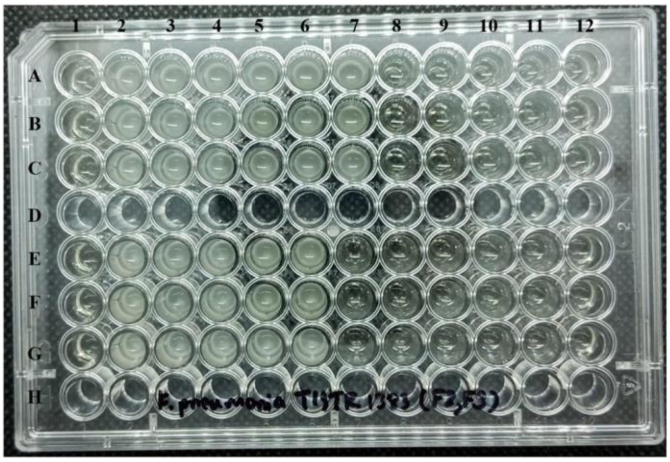

Figure S7. MIC of Col-AgNPs in F2 and F3 against *K. pneumonia* TISTR 1383. Blank (wells 1A-1C and 1E-1G), untreated (wells 2A-2C and 2E-2G), Col-AgNPs in F2 at 0.25, 0.5, 1, 2, 4, 8, 16, 32, 64, and 128 µg/mL (wells 3A-3C – 12A-12C, respectively), and Col-AgNPs in F3 at 0.25, 0.5, 1, 2, 4, 8, 16, 32, 64, and 128 µg/mL (wells 3E-3G – 12E-12G, respectively).

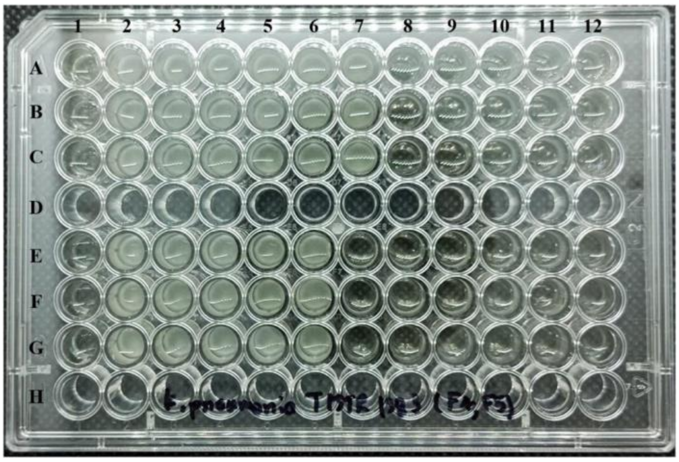

Figure S8. MIC of Col-AgNPs in F4 and F5 against *K. pneumonia* TISTR 1383. Blank (wells 1A-1C and 1E-1G), untreated (wells 2A-2C and 2E-2G), Col-AgNPs in F4 at 0.25, 0.5, 1, 2, 4, 8, 16, 32, 64, and 128 µg/mL (wells 3A-3C – 12A-12C, respectively), and Col-AgNPs in F5 at 0.25, 0.5, 1, 2, 4, 8, 16, 32, 64, and 128 µg/mL (wells 3E-3G – 12E-12G, respectively).

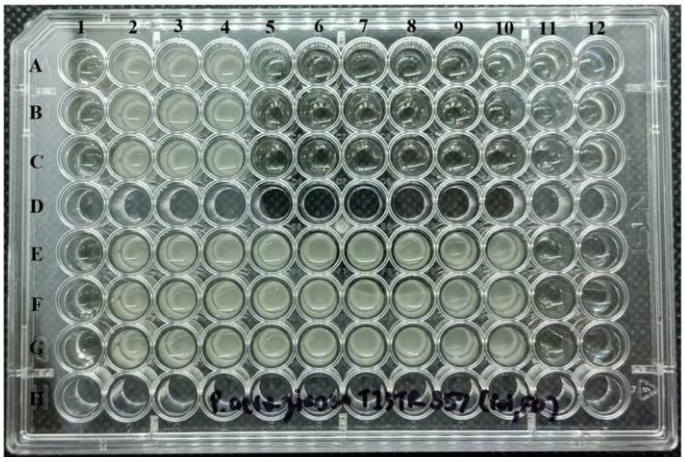

Figure S9. MIC of colistin and AgNPs in F0 against *P. aeruginosa* TISTR 357. Blank (wells 1A-1C and 1E-1G), untreated (wells 2A-2C and 2E-2G), colistin at 0.25, 0.5, 1, 2, 4, 8, 16, 32, 64, and 128 μg/mL (wells 3A-3C – 12A-12C, respectively), and AgNPs in F0 at 0.25, 0.5, 1, 2, 4, 8, 16, 32, 64, and 128 μg/mL (wells 3E-3G – 12E-12G, respectively).

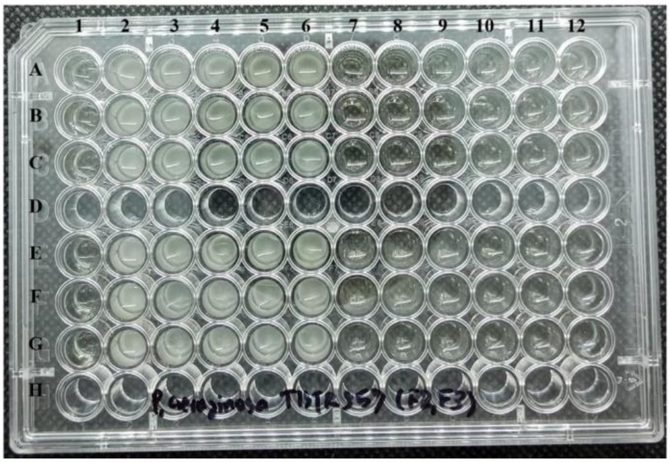

Figure S10. MIC of Col-AgNPs in F2 and F3 against *P. aeruginosa* TISTR 357. Blank (wells 1A-1C and 1E-1G), untreated (wells 2A-2C and 2E-2G), Col-AgNPs in F2 at 0.25, 0.5, 1, 2, 4, 8, 16, 32, 64, and 128 μg/mL (wells 3A-3C – 12A-12C, respectively), and Col-AgNPs in F3 at 0.25, 0.5, 1, 2, 4, 8, 16, 32, 64, and 128 μg/mL (wells 3E-3G – 12E-12G, respectively).

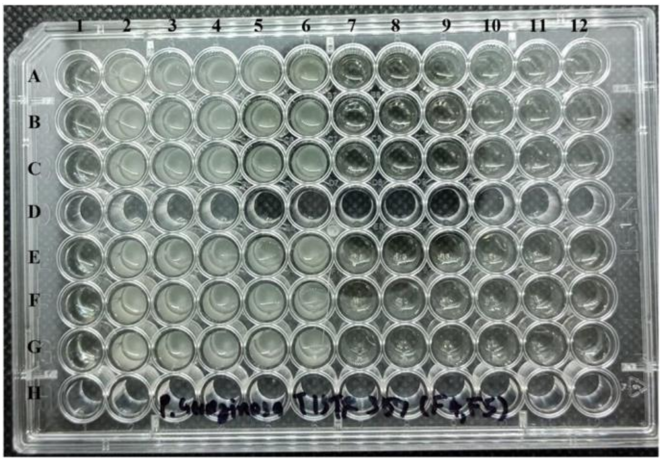

Figure S11. MIC of Col-AgNPs in F4 and F5 against *P. aeruginosa* TISTR 357. Blank (wells 1A-1C and 1E-1G), untreated (wells 2A-2C and 2E-2G), Col-AgNPs in F4 at 0.25, 0.5, 1, 2, 4, 8, 16, 32, 64, and 128 μg/mL (wells 3A-3C – 12A-12C, respectively), and Col-AgNPs in F5 at 0.25, 0.5, 1, 2, 4, 8, 16, 32, 64, and 128 μg/mL (wells 3E-3G – 12E-12G, respectively).

Table S1. Summary of the parameters from the validated HPLC method for determining colistin in Col-AgNPs.

Parameters	Results
Range	2 - 150 μg/mL
Linearity (R^2)	0.9997
LOD	0.56 μg/mL
LOQ	1.70 μg/mL
Precision (%RSD)	0.50
Accuracy (%Recovery)	99.09 ± 0.82
50% level	98.83 ± 0.34
100% level	99.68 ± 1.16
150% level	98.76 ± 0.64
